# Pleomorphic hyalinizing angiectatic tumor of the vulva: literature review based on a rare presentation

**DOI:** 10.4322/acr.2021.353

**Published:** 2022-02-11

**Authors:** Eveline Cristina da Silva, Rodrigo Fonseca Abreu, Antônio Geraldo Nascimento, Louise De Brot Andrade

**Affiliations:** 1 AC Camargo Cancer Center, Department of Anatomic Pathology, São Paulo, SP, Brasil

**Keywords:** Soft Tissue Neoplasms, Vulvar Neoplasms, Sarcoma

## Abstract

Pleomorphic hyalinizing angiectatic tumor (PHAT) of soft tissues is a rare, non-metastatic tumor of unknown etiology and uncertain behavior, which may recur locally. There are few reports on this condition, and due to the rarity of the disease, its lineage has not yet been fully elucidated. The present study aims to report the case of an unusual entity observed for the first time in vulval topography. A female patient, 83 years old, presented with a tumor in the vulvar region that had evolved for approximately 4 months. Magnetic resonance imaging showed an expansive perineal formation of 8.5 × 3.5 cm, and a hemivulvectomy with a flap rotation was performed. The review of the slides revealed a mesenchymal lesion without significant atypia, which was richly vascularized. In the areas of interest, the immunohistochemical (IHC) study demonstrated positivity for CD34, estrogen, and progesterone receptors; it was negative for the other tested markers. Morphological findings associated with the IHC staining panel supported the diagnosis of PHAT. The main morphological features of PHAT are clusters of ectatic vessels of different sizes that show deposits of subendothelial and intraluminal fibrin. Fusiform and pleomorphic cells randomly arranged in leaves or long fascicles intermingle these vessels. It is essential to recognize this entity and consider it among the differential diagnoses of a mesenchymal lesion, given the wide variety of entities that comprise this group of lesions.

## INTRODUCTION

In 1996, Smith et al.^1^ first reported the pleomorphic hyalinizing angiectatic tumor (PHAT) of soft tissue tumor of unknown etiology and uncertain behavior, which may recur locally. It affects patients with a mean age of 50 years, ranging from the first to the eighth decade of life, with no predilection for sex. It occurs as a painless, slow-growing mass and is often found in the subcutaneous tissue of the lower extremities, especially in the ankle and foot, and rarely presents as a deep soft tissue mass.^2^^,^ 3

Due to the rarity of the disease, its lineage has not yet been fully elucidated with scarce published reports. The clinical behavior of PHAT is characterized by local recurrence in up to 50% of cases, but metastases have not been documented to date.^2^^-^ 5 Local excision with wide margins is recommended as the best therapeutic approach whenever possible, as this tumor has a local recurrence rate of around 33%.^4^^,^ 6 ^,^ 7 There is no relevant evidence for the role of adjuvant therapy in PHAT.^5^^,^ 7 Adjuvant radiotherapy has been used in some case series to reduce the rate of local recurrence, but more evidence is needed before indicating its use, and chemotherapy is not useful in view of the absence of metastases.^5^^,^ 7

Since its original morphological description, attention has focused on the lesion’s striking vascular patterns, distinguishing PHAT from other mesenchymal lesions.^4^ PHAT is characterized by dilated blood vessels with prominent circumferential hyalinization; it is surrounded by a cell proliferation composed of spindle-shaped and pleomorphic cells, some with hemosiderin, a low mitotic index, and variable inflammatory component.^1^^,^ 2

Mesenchymal lesions constitute a heterogeneous group of alterations, represented by many distinct entities with overlapping morphological patterns, thus challenging the pathologist. This study aims to report the case of an unusual entity, observed for the first time in vulval topography, highlighting its importance among the differential diagnoses of mesenchymal lesions, emphasizing the morphological aspect of this entity as a key factor for the diagnosis.

## CASE REPORT

**Figure 1:**
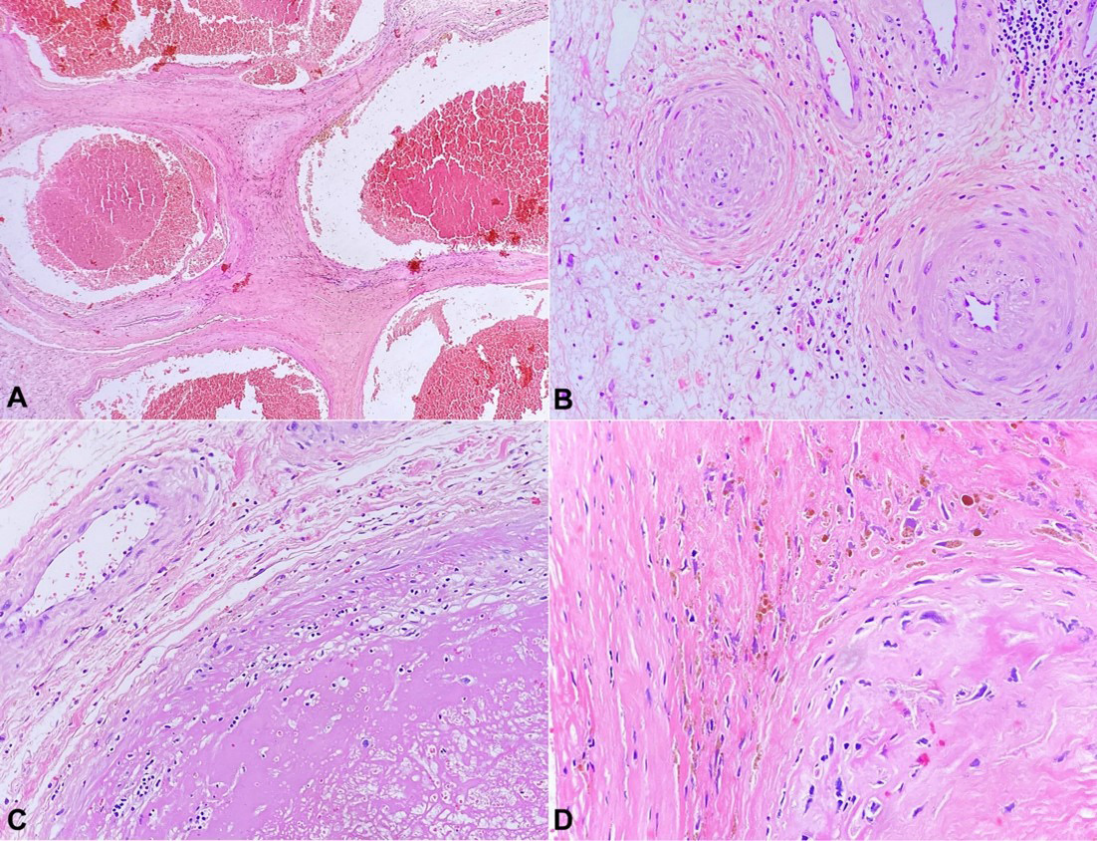
Photomicrographs of the surgical specimen showing variably sized ectatic vessels with hyalinization (A and B) (H&E, 40X), sometimes containing thrombi in organization (C) (H&E, 100X), intermixed with myxoid and edematous stroma and intracytoplasmic hemosiderin deposits (D) (H&E, 100X).

**Figure 2:**
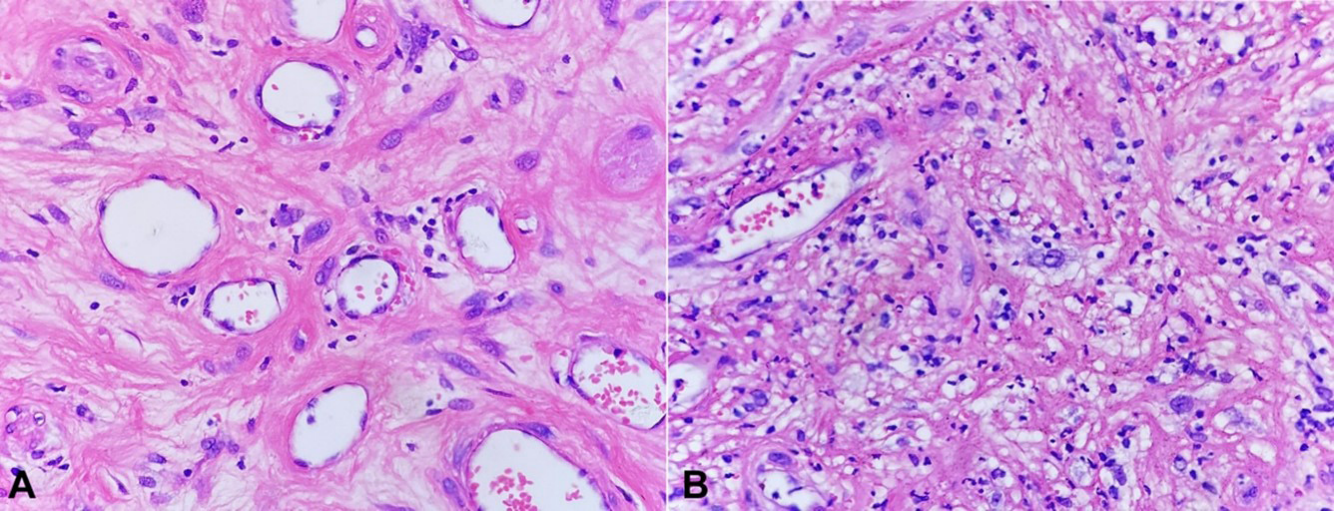
Photomicrographs of the surgical specimen showing in A and B scattered spindle-shaped and rounded tumor cells with pleomorphic nuclei (H&E,400X). Presence of pleomorphic spindle cells with intranuclear inclusions (A); mitotic figures are absent. An infiltrate of mixed chronic inflammatory cells is usually present, with a predominance of lymphocytes (B).

An 83-year-old female patient sought medical care at another service reporting the presence of a tumor in the vulvar region with approximately over the last 4 months Her medical history included dyslipidemia, type 2 diabetes mellitus, and hypothyroidism. The abdominopelvic magnetic resonance imaging was requested, which showed an expansive perineal formation of 8.5 × 3.5 cm, and the patient was referred for surgical resection: a hemivulvectomy with flap rotation. The material was sent to the external pathological anatomy service, which concluded—as a morphological diagnosis—a fibroepithelial lesion measuring 4.2 cm in diameter, with free margins. The patient was referred to our institution for follow up with the histopathological material for a second look by another team of pathologists. The conclusion was a mesenchymal lesion without significant atypia, which was richly vascularized (Figure 1 and 2).

**Figure 3:**
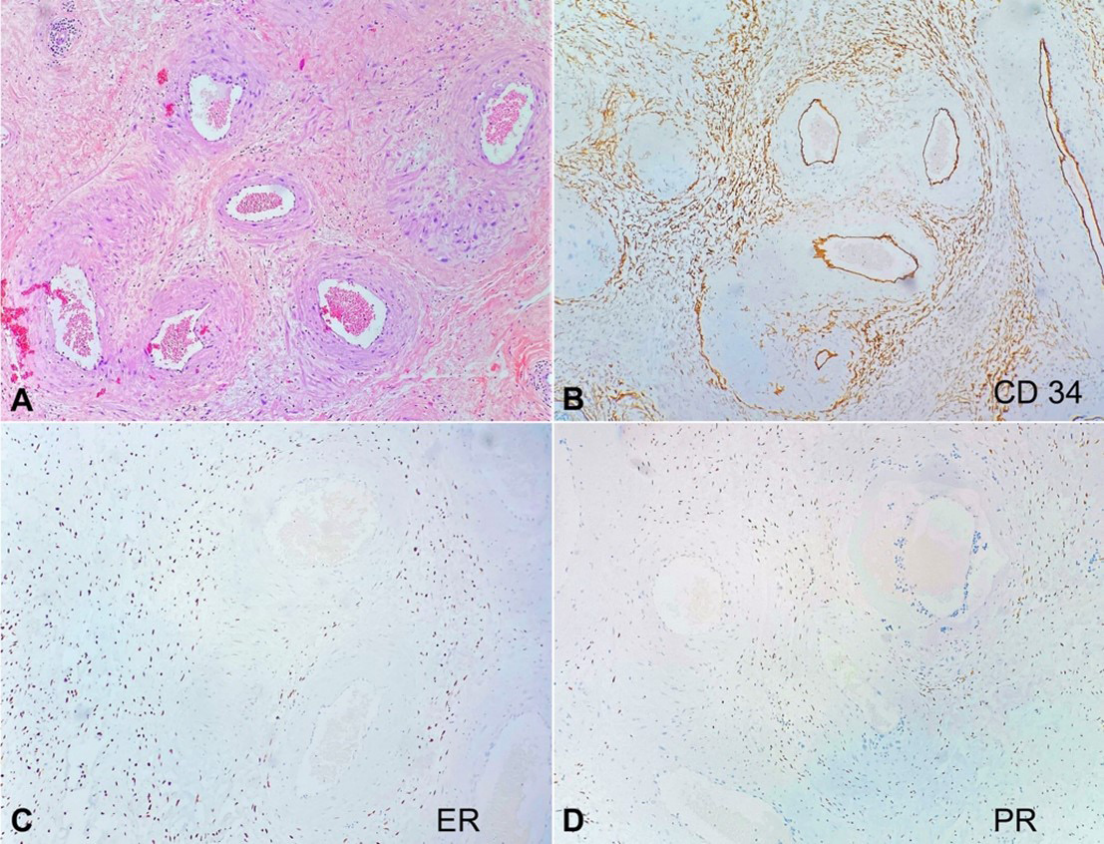
Photomicrograph of the surgical specimen. In (A) a representative field of the lesion in hematoxylin-eosin staining was chosen (40X). Sequentially, in immunohistochemistry, tumor cells were positive for CD34 (B), ER (C) and PR (D)

**Figure 4:**
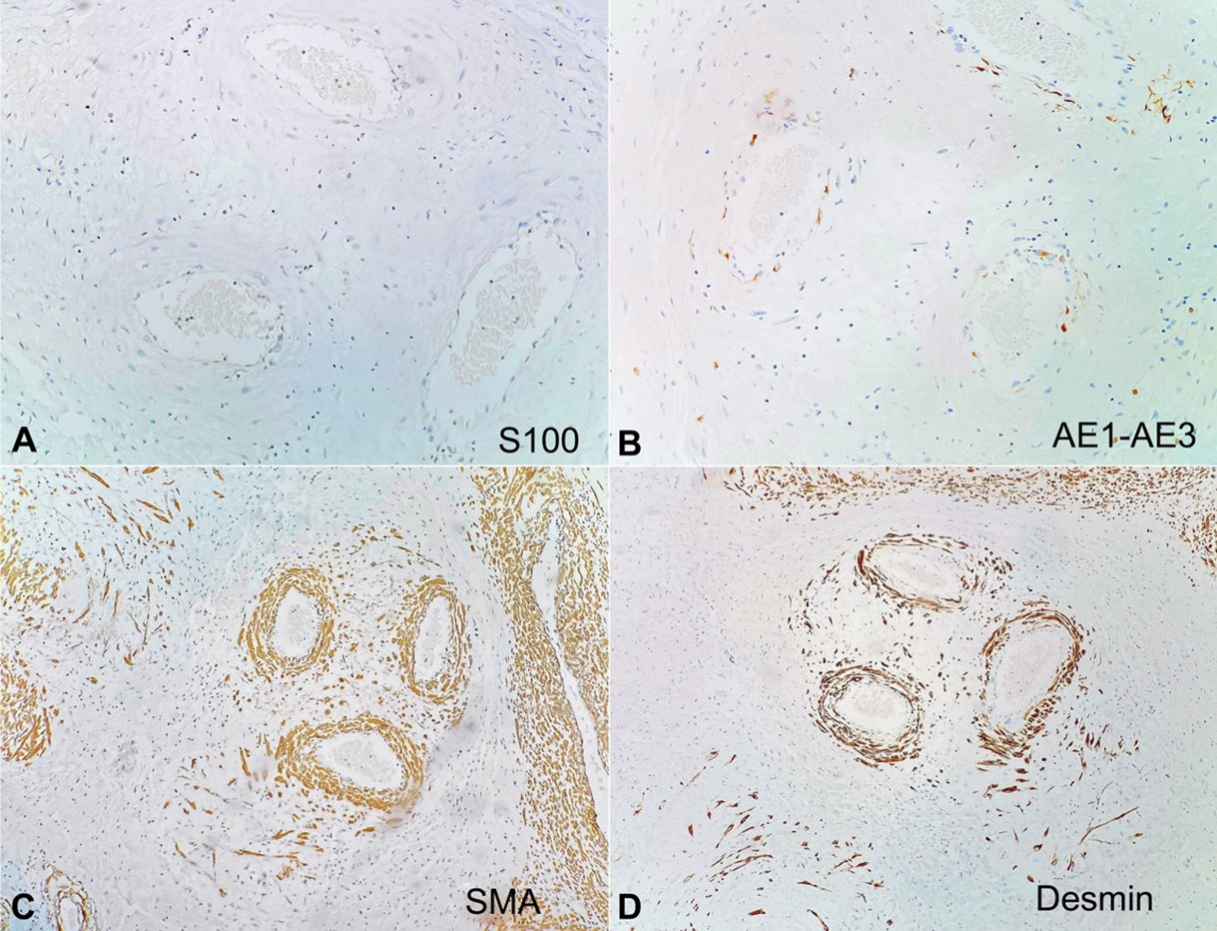
Photomicrograph of the surgical specimen showing negativity for protein S-100 (A), AE1/AE3 (B), SMA (C) and desmin (D).

In the areas of interest, the immunohistochemical (IHC) study demonstrated positivity for CD34, estrogen and progesterone receptors, and was negative for cytokeratin AE1/AE3, desmin, smooth muscle actin, and protein S100 (Figure 3 and 4). Morphological findings associated with the IHC staining panel supported the diagnosis of PHAT. The patient is in therapeutic follow-up currently, with no recurrence of the lesion and metastasis.

## DISCUSSION

PHAT is an adult soft tissue neoplasm located mainly in the subcutaneous tissue of the lower extremities. It was first described by Smith et al.,^1^ who analyzed 14 cases among men and women with no predilection for gender and mean patient age of 50 years; the study suggested a single neoplasm with a low degree of malignancy.^1^ The World Health Organization (WHO) defines the condition as “very rare,” with around 100 cases reported in the world literature.^5^^,^ 6

There are references in articles that described PHAT in other topographies, which include the trunk, upper extremities, axilla, inguinal region, perineum, buttocks, back, oral cavity, and scrotum.^5^^,^ 7 In a systematic search using the PubMed search tool, 52 articles that allude to PHAT were found in 118 reported cases (Table 1). To date, this is the first case of PHAT described in the vulvar region.

**Table 1:** List of published studies of PHAT

Ref	Sex/Age (y)	Tumor location	Ref	Sex/Age (y)	Tumor location	Ref	Sex/Age (y)	Tumor location
1	M/33	Shoulder	4	F/46	Leg	8	F/58	Foot
	F/78	Leg		F/77	Thigh		M/33	Thigh
	F/62	Chest wall		F/79	Foot	9	F/37	Ankle
	F/33	Ankle		F/77	Thigh	10	M/46	Leg
	M/70	Thigh		M/67	Arm		F/49	Foot
	F/32	Leg		M/89	Leg		F/87	Arm
	F/59	Thigh		M/74	Buttock	11	F/71	Renal Hilum
	M/72	Thigh		F/75	Thigh	12	F/35	Neck
	M/60	Thigh		M/49	Inguinal	13	F/63	Leg
	F/58	Chest wall		M/63	Inguinal	14	F/68	Thigh
	F/78	Arm		F/38	Back	15	M/76	Arm
	M/57	Buttock		F/51	Foot	16	M/62	Leg
	M/83	Thigh		M/32	Arm	17	F/51	Chest wall
	F/51	Ankle		M/44	Buttock	18	F/55	Foot
19	M/58	Axilla		F/67	Ankle	20	M/56	Thigh
21	F/88	Thigh		F/59	Leg	22	F/26	Pelvis
23	F/59	Foot		F/42	Foot	24	M/22	Forearm
25	F/31	Right Foot		M/43	Leg	26	M/35	Hand
27	F/41	Leg	28	M/69	Back	29	F/79	Inguinal
	F/44	Leg	30	F/86	Buccal mucosa	31	F/61	Renal
32	M/63	Forearm	33	F/45	Thigh	34	M/50	Left Calf
35	F/83	Thigh	36	M/63	Breast	37	M/50	Right Buttock
4	F/39	Hand	38	F/60	Foot		M/72	Right Buttock
	F/30	Ankle	39	M/66	Buttock	40	F/63	Left Crura
	M/42	Forearm	41	M/76	Thigh	7	F/30	Pelvis
	F/46	Foot	42	F/76	Axilla	43	M/45	Left Arm
	M/10	Foot	44	F/77	Flank	45	F/74	Right Popliteal Fossa
	F/51	Ankle	46	F/30	Right Inguinal	47	F/33	Cheek
	F/44	Ankle		M/76	Left Inguinal	5	F/48	Right Thigh
	M/67	Leg		F/50	Right Waist			
	F/32	Leg		M/37	Right Thigh			
	M/36	Thigh		M/67	Left Thigh			
	F/50	Ankle		F/33	Right Forearm			
	F/53	Foot		F/19	Right Buttock			
	F/84	Leg		M/52	Left Foot			
	F/57	Foot		F/47	Right Chest wall			
	F/49	Leg	48	F/53	Mesorrectum			
	M/56	Axilla	49	M/75	Breast			
	M/49	Thigh	50	F/65	Forearm			
	M/79	Pelvis	6	M/49	Buttock			
	M/60	Ankle	51	M/46	Leg			
	M/27	Thigh	52	M/68	Scrotum			
4	M/66	Ankle	53	F/37	Foot			
	M/54	Foot	54	M/53	Right Groin			
	F/32	Leg	8	F/58	Foot			

F = female; M = male; Ref = reference; y = year.

The main morphological characteristics of PHAT are clusters of ectatic vessels of different sizes that show deposits of subendothelial and intraluminal fibrin.^1^^,^ 4 ^,^ 9 ^,^ 53 ^,^ 55 ^-^ 58 Fibrin organization creates prominent perivascular collagen cuffs, which, in some tumors, give rise to large areas of stromal hyalinization. Among these vessels are fusiform and pleomorphic cells, which are sometimes foamy, round, and are randomly arranged in leaves or long fascicles.^1^^-^ 4 ^,^ 6 ^,^ 55 Pleomorphic cells contain intranuclear pseudoinclusions (sometimes easily identifiable), finely granular cytoplasmic hemosiderin (located mainly in cells adjacent to vessels), and inflammatory cells (predominantly lymphocytes, plasma cells, and mast cells).^1^^-^ 4 ^,^ 6 ^,^ 55 These essential features for morphological diagnosis were found in our case.

The most prominent morphological characteristic in PHAT is the hyalinizing angiectatic vascular structure.^1^^,^ 4 ^,^ 6 However, its meaning is still not fully understood. Smith et al.^1^ proposed that hyalinized vessels arise as a consequence of the gradual invasion of tumor cells into normal vessels in the topography, which could result in endothelial damage and consequent exudation of plasma components, leading to the formation of perivascular hyaline deposits. However, this does not cause massive vascular destruction with tumor necrosis, which usually occurs in tumors with a high degree of malignancy.^6^ Another cause for this vascular deposition is related to the presence of mast cells that release vasoactive substances resulting from the response to tissue damage suffered by tumor infiltration; this results in increased vascular permeability and consequent perivascular hyaline deposition.^1^^,^ 27

In an attempt to immunologically characterize these neoplasms, a variety of IHC markers have been used.^59^ More consistently in the literature, expressions of CD34, factor XIIIa, vascular endothelial growth factor (VEGF), and CD99 were found, and the absence of any expression of S100, CD68, CD31, desmin, smooth muscle actin, and epithelial markers was noted.^9^^,^ 18 ^,^ 35 ^,^ 37 ^,^ 38 ^,^ 42 ^,^ 44

CD34 is present in approximately 70% of tumors; the theoretical basis is tumor histogenesis, which consists of tumor cells derived from fibroblasts of the microvascular adventitia.^23^ Factor XIIIa has focal positivity and is present in 20%–40% of tumors. However, its interpretation is still controversial as it is not known whether the immunostaining is in fact in tumor cells or in non-neoplastic stromal cells present in the middle of the tumor.^23^^,^ 27

Groisman et al.^27^ introduced the premise of tumor angiogenesis as an intrinsic mechanism of PHAT—associated with the presence of VEGF—and studied its immunostaining in their cases, verifying the positivity in endothelial cells of non-hyalinized vessels, located mainly in the periphery of the tumor and in the tumor cells. In contrast, there was negativity in the endothelium of the hyalinized vessels. Taking up the ideas of Smith et al.^1^ regarding the mechanism of perivascular collagen deposition, it is suggested that this hyalinization—regardless of the origin of the mechanism—leads to progressive vascular thrombosis with consequent focal hypoxia and necrosis, triggering again the release of VEGF by the cells’ tumors and consequently stimulating angiogenesis once more.^1^

Other immunomarkers, such as CD99, were mentioned in the literature, with variable marking, and without diagnostic relevance.^27^^,^ 39 ^,^ 49 ^,^ 60 The need for an IHC panel with negative markers, such as S100, AE1/AE3, desmin, CD31, and CD68, aims to help in the differential diagnosis in relation to other mesenchymal and non-mesenchymal tumors.^23^^,^ 27 ^,^ 59

Our case showed positivity for CD34, estrogen and progesterone receptors, and negativity for cytokeratin AE1/AE3, desmin, smooth muscle actin, and protein S100 (Figure 2). CD34—associated with positive hormone receptors—is related to the appearance of the tumor in the vulvar region, which is characteristic of some of the mesenchymal tumors in this topography that arise from superficial, hormonally responsive stromal cells of the lower genital tract. This corroborates with the lesion, if present, as a primary mesenchymal tumor of the vulva.^61^

The main differential diagnoses found in the searches are schwannoma, pleomorphic sarcomas (formerly called malignant fibrous histiocytoma), solitary fibrous tumor (SFT) and giant cell angiofibroma.^1^^,^ 18 ^,^ 19 ^,^ 34 ^,^ 62 ^,^ 63 Tumor cells resemble those of pleomorphic sarcomas, but differ from them by the presence of prominent intranuclear cytoplasmic pseudoinclusions, the scarcity of mitotic figures, and the frequent presence of CD34 expression.^1^^,^ 59 These tumors also share several characteristics with schwannomas, such as their hyalinized vessel wall, intranuclear cytoplasmic inclusions, very low mitotic activity, and the presence of mast cells; however, they are differentiated by the frequent presence of infiltrating margins and the absence of S-100 protein labeling.^1^^,^ 60

PHAT shares morphological characteristics with SFT and giant cell angiofibroma.^1^^,^ 19 ^,^ 34 ^,^ 59 Nuclear atypia found in PHAT are more prominent than those seen in SFT, and the presence of multinucleated giant cells is seen only in PHAT.^1^^,^ 19 ^,^ 34 ^,^ 59 Prominent clusters of thin-walled ectatic vessels surrounded by perivascular hyaline material are characteristic in PHAT, but may also be present in solitary fibrous tumors and giant cell angiofibromas, and may correspond to secondary changes due to circulation disorders often seen in tumors of slow growth.^1^^,^ 19 ^,^ 34 ^,^ 59

Two other entities mentioned in the literature are relevant for discussion about the morphological diagnosis of PHAT: hemosiderotic fibrolipomatous tumor (HFLT) and myxoinflammatory fibroblastic sarcoma (MIFS).^2^^,^ 4 ^,^ 53 ^,^ 56 ^-^ 58 ^,^ 62 ^,^ 63

Many PHATs demonstrate stratified peripheral zones of HFLT, and PHAT-like foci are frequently present in HFLT.^4^^,^ 53 ^,^ 56 ^-^ 58 In addition, cytogenetic studies demonstrate genetic rearrangements represented by breakpoints within the transformer-receptor growth factor 3 (TGFBR3) genomic loci on chromosome 1p22 and meningioma-expressed antigen 5 (MGEA5) on chromosome 10q24 that have been identified in substantial subsets of PHAT, HFLT, and MIFS.^4^^,^ 53 ^,^ 56 ^-^ 58 Tumors still showing histological overlap between MIFS and HFLT/PHAT, and the same genetic alterations described above, have also been reported, suggesting a link between these three entities. However, this remains controversial and is not fully understood, leading to the most recent WHO international classification of soft tissue tumors, which maintains them as three distinct entities.^2^^,^ 4 ^,^ 53 ^,^ 56 ^-^ 58

However, based on their overlapping clinical, morphological, and genetic characteristics, PHAT, HFLT, and MIFS may represent a family of closely related lesions or different morphological manifestations of a single entity, characterized by a predilection for the distal extremity, locally aggressive behavior, and very low metastatic potential.^2^^,^ 4 ^,^ 53 ^,^ 56 ^,^ 57

Despite sharing the characteristics of HFLT/PHAT, the presence of metastasis was reported in only a few articles regarding MIFS.^5^^,^ 62 Even given the small number of reported cases, this finding should be considered in the follow-up of these patients, especially if there is a recurrence.^5^^,^ 62 More studies are needed to elucidate the pathogenesis and biological potential of these entities.

## CONCLUSION

PHAT is a soft tissue tumor with low-to-intermediate malignancy potential, sharing histological similarities with benign and low-grade malignant tumors. Therefore, recognizing this entity and placing it among the differential diagnoses facing a mesenchymal lesion is essential, given the wide variety of entities that comprise this group of lesions.

Within the literature review presented, PHAT was not found as a primary lesion of the vulva. Our case’s morphology and immunohistochemistry are remarkably similar to those found in PHAT from other topographies, with the peculiarity of positive hormone receptors that can infer the diagnosis of primary mesenchymal tumor in the vulvar region.

In this context, the histopathological aspects are essential for the diagnosis of this lesion, as well as the appropriate therapeutic management for each patient. This requires the recognition of morphological criteria, proper interpretation of IHC and cytogenetic studies—when requested—and the association of these findings with clinical data for accuracy in the analysis of this tumor.
